# The Ddx5 and Ddx17 RNA helicases are cornerstones in the complex regulatory array of steroid hormone-signaling pathways

**DOI:** 10.1093/nar/gkt1216

**Published:** 2013-11-25

**Authors:** Samaan Samaan, Léon-Charles Tranchevent, Etienne Dardenne, Micaela Polay Espinoza, Eleonora Zonta, Sophie Germann, Lise Gratadou, Martin Dutertre, Didier Auboeuf

**Affiliations:** ^1^Université de Paris Diderot-Paris 7, F-75013 Paris, France, ^2^Inserm U1052, F-69008 Lyon, France, ^3^CNRS UMR5286, F-69008 Lyon, France, ^4^Centre de Recherche en Cancérologie de Lyon, 69008 Lyon, France and ^5^Université de Lyon 1, F-69100 Villeurbanne, France

## Abstract

Estrogen and androgen receptors (ER and AR) play key roles in breast and prostate cancers, respectively, where they regulate the transcription of large arrays of genes. The activities of ER and AR are controlled by large networks of protein kinases and transcriptional coregulators, including Ddx5 and its highly related paralog Ddx17. The Ddx5 and Ddx17 RNA helicases are also splicing regulators. Here, we report that Ddx5 and Ddx17 are master regulators of the estrogen- and androgen-signaling pathways by controlling transcription and splicing both upstream and downstream of the receptors. First, Ddx5 and Ddx17 are required downstream of ER and AR for the transcriptional and splicing regulation of a large number of steroid hormone target genes. Second, Ddx5 and Ddx17 act upstream of ER and AR by controlling the expression, at the splicing level, of several key regulators of ER and AR activities. Of particular interest, we demonstrate that Ddx5 and Ddx17 control alternative splicing of the GSK3β kinase, which impacts on both ER and AR protein stability. We also provide a freely available online resource which gives information regarding splicing variants of genes involved in the estrogen- and androgen-signaling pathways.

## INTRODUCTION

The sex steroid hormones, estrogen and testosterone, influence normal physiology, reproduction and behavior. Their biological functions are mediated through cognate nuclear receptors that govern gene expression in hormone-sensitive tissues. Many lines of evidence have implicated steroid hormones as etiologic factors in the origin and progression of various malignancies (1,2). Perturbation of the estrogen-signaling pathway is associated with two-thirds of breast cancers that express the estrogen receptor alpha (ERα), which is considered as a good prognosis marker. The androgen receptor (AR) is activated by the binding of testosterone or its physiologically active metabolite, 5α-dihydrotestosterone (DHT) and is involved in prostate cancer initiation and metastasis.

ERα and AR are members of the large superfamily of nuclear receptors and act as ligand-activated transcription factors. The canonical model of steroid receptor action implies a ligand-specific conformational change triggering its phosphorylation, homodimerization and binding to hormone responsive elements located in promoters or regulatory regions of target genes (3). Activation of the estrogen and androgen pathways requires the concerted action of a plethora of factors. Some of them are involved in posttranslational modifications of the hormone receptors, impacting for example on their subcellular localization or stability (4–6). Other factors, the so-called transcriptional coregulators, are recruited by hormone receptors on target promoters to mediate their effects on transcription (7,8). For both ERα and AR, dozens of coregulators, including coactivators and corepressors, have been identified. However, most of them have been studied in the context of a few target genes, and therefore it is currently unknown whether they contribute to the hundreds of gene regulations induced by hormones.

Among these, the DEAD-box RNA helicase Ddx5 (p68) and its highly related paralog, Ddx17 (p72), are transcriptional coregulators of ERα and AR (9,10). Ddx5 and Ddx17 interact directly with ERα and AR and were shown in a few cases to be recruited to target promoters (11,12) where they might modulate RNA polymerase II recruitment. However, the extent of ERα and AR endogenous target genes on which Ddx5/Ddx17 act as transcriptional coregulators is not known. In addition to being transcriptional coregulators, Ddx5 and Ddx17 are *bona fide* components of the splicing machinery, the spliceosome (13), and play a role in the regulation of alternative splicing that leads to the production from the same gene of several splicing variants coding for different protein isoforms with different and sometimes opposite biological activities (14–17). Alternative splicing is the rule, not the exception, as 90% of human genes produce several splicing variants. Alternative splicing is the main mechanism increasing the diversity of the proteome coded by a limited number of genes (18). In this context, the Ddx5 and Ddx17 multifunctional proteins could coordinate transcription and splicing allowing the production of the proper isoform from hormone target genes as previously suggested by using minigene reporter assay (19). However, whether Ddx5 and Ddx17 regulate splicing of endogenous hormone target genes is not known.

In this work, we report using large-scale approaches for first time that Ddx5 and Ddx17 are master regulators of the estrogen and androgen-signaling pathways. Indeed, these proteins are not only required for regulating the expression of a large number of endogenous estrogen- and androgen-target genes both at the transcriptional and splicing level but remarkably, they are also acting upstream of the estrogen and androgen receptors by controlling the expression, at the splicing level, of several key regulators of the hormone-signaling pathways.

## MATERIALS AND METHODS

### Cell culture and stable cell lines

MCF-7 cells were grown in DMEM and LNCaP cells in RPMI-1640 (Gibco/LifeTechnologies). Both mediums were supplemented with 10% fetal bovine serum, 1% glutamine and 1% penicillin/streptomycin. Cells were maintained at 37°C in a humidified atmosphere of 95% air and 5% CO_2_. Wild-type Ddx5-HA and Ddx17-HA and mutated Ddx5-HA K144A and Ddx17-HA K142R were cloned into pTRE2-hyg vectors to generate inducible MCF-7/Tet-On stable cell lines (Clontech). Resistant clones were selected with hygromycin (300 mg/ml, Clontech) and protein expression was checked after Doxycycline treatment (1 µg/ml) for 48 h.

### Cell transfection and treatment

A total of 3 × 10^6^ cells were transiently transfected with 26.6 nM siRNAs (Supplementary Table S3) using Lipofectamine RNAiMax (LifeTechnologies). Twenty-four hours before treatment, MCF-7 and LNCaP cells, were cultured in red phenol-free medium supplemented with 2% charcoal-treated FBS. Cells were treated with E2 (10 nM; Sigma) for 1 or 10 h or DHT (10 nM; Sigma) for 24 h. Control cells received equal volumes of vehicle (ethanol).

### RNA preparation, RT–PCR and RT–qPCR

Total RNAs were prepared using TRIpure Isolation Reagent (Roche), and 1 µl of Glycoblue (Ambion) was added before RNA precipitation. Nuclear fractionation was performed as previously described (20). Reverse transcription (RT) was performed with 1–2 µg of total RNA using M-MLV Reverse Transcriptase (LifeTechnologies) and random primers. The RT reactions were diluted and used either for PCR analysis using GoTaq Flexi DNA Polymerase (Promega) or in qPCR using SYBR Green I Master Mix (Roche) on a Roche LightCycler 480 II. Primer sequences are provided in Supplementary Table S3. The relative RNA levels were determined on the basis of the threshold cycle (*C*_t_) for each qPCR product and normalized to 18S ribosomal RNA levels.

### Affimetrix exon array

One microgram of total RNA was processed with the GeneChip WT Sense Target Labeling kit and hybridized to GeneChip Human Exon 1.0 ST arrays. Affymetrix exon-array data were normalized with quantile normalization. Antigenomic probes were used to perform the background correction. Only probes targeting exons annotated from full-length cDNA were retained for analysis. Cross hybridizing probes and probes with lower signals intensity than anti-genomic background probes showing the same GC content were removed. Only probes with a DABG *P*-value ≤ 0.05 in at least half of the arrays were considered for further statistical analysis. Arrays were performed in four independent replicates. The strategies adopted to identify E2-, DHT-regulated and Ddx5/17-dependent genes are described in Supplementary Figure S1. Briefly, the median intensity of all constitutive exonic probes was calculated for each gene in each sample, and the experimental samples and control groups were compared using a Student’s paired *t*-test. Paired statistical analyses were performed using the Student’s paired *t* test on the splicing index (SI) to analyze the Exon Array data at the exon level (SI > 1.5, *P* < 0.05). The SI corresponds to a comparison of gene-normalized exon intensity values between the two analyzed experimental conditions.

### Western blot analysis

Total protein extracts were obtained using NP-40 buffer (50 mM Tris–HCl pH 8, 0.4 M. NaCl, 5 mM EDTA pH 8, 1% NP40, 0.2% SDS, 1 mM DTT) supplemented with Protease and Phosphatase Inhibitors (Roche). An amount of 20 µg of proteins were separated by NuPAGE® Novex 3-8% Tris-Acetate or 12% bis-tris-Acetate Gels (LifeTechnologies). Membranes were incubated with specific primary antibodies against Ddx5 (ab10261, Abcam), Ddx17 (ab24601 Abcam), SMRT (H-300, sc-20778 Santa Cruz), AR (441, sc-7305), ERα (F-10, sc-8002 Santa Cruz), GSK3β (27C10, Cell signaling) and β-Actin (I-19, sc-1616 Santa Cruz).

### Chromatin-immunoprecipitation

ERα chromatin-immunoprecipitation (ChIP) assay was performed in MCF-7 cells using anti-ERα antibody (HC-20, sc-543 Santa Cruz). Ddx5-HA ChIP assay was performed in inducible MCF-7 cell line as previously described (21) using anti-HA antibody (3F10, Roche). MCF-7 or MCF-7 inducible cell lines were maintained in red phenol-free medium supplemented with 2% charcoal-treated FBS. Cells were treated with E2 (10 nM; Sigma) for 1 h before fixation. Primers used are detailed in Supplementary Table S3.

## RESULTS

### Ddx5 and Ddx17 are master regulators of the estrogen-signaling pathway

**Figure 1.**
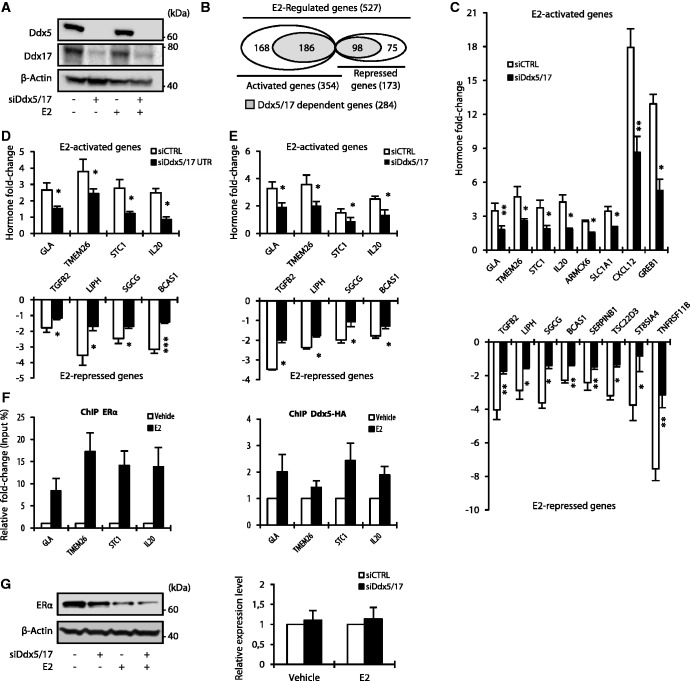
Ddx5 and Ddx17 are master regulators of the estrogen-signaling pathway. (**A**) Western blot analysis of Ddx5, Ddx17 and β-Actin as loading control, using total protein extract from MCF-7 cells transfected with either a control siRNA or siDdx5/17 in the presence or absence of estradiol (E2) for 10 h. (**B**) *In silico* prediction of Exon array analysis of genes regulated at the global expression level by E2 and dependent of Ddx5 and Ddx17 (in gray). (**C**) Hormone fold change induced by E2 treatment for 10 h of the expression level of a subset of mRNAs as determined by RT–qPCR in MCF-7 cells transfected with siCTRL or siDdx5/17 (genes upregulated by E2 in the upper panel and genes downregulated by E2 in the lower panel). (**D**) Hormone fold change induced by E2 treatment for 10 h of the expression level of a subset of mRNAs as determined by RT–qPCR in MCF-7 cells transfected with siCTRL or a mixture of siRNAs-targeting Ddx5 and Ddx17 UTRs (siDdx5/17 UTR). (**E**) Hormone fold change as assessed by RT-qPCR measuring the unspliced pre-mRNAs of E2-regulated genes in the nuclear extracts. MCF-7 cells were transiently transfected with Control siRNA or siDdx5/17 then treated with E2 or vehicle for 1 h. (**F**) qPCR analysis using primers spanning promoter regions of a subset of E2-regulated genes on genomic DNA in a ChIP assay using MCF-7 for ERα (left panel) and MCF-7 stable cell lines expressing Ddx5-HA (right panel). (**G**) Western blot analysis of ERα and β-Actin as loading control using MCF-7 cells transfected with either a control siRNA or siDdx5/17 in the presence or absence of estradiol (E2) for 10 h (left panel). Relative expression level of ERα mRNA as assessed by RT–qPCR analysis (right panel). Histograms represent the average of at least three independent experiments. Error bars, s.e.m.; *P < 0.05; **P < 0.01; ***P < 0.001 (*t*-test).

In order to assess the extent to which Ddx5 and Ddx17 participate in gene expression regulation in response to estradiol (E2), ER-positive MCF-7 breast cancer cells were treated with E2 or vehicle for 10 h after being transfected with a control siRNA (siCTRL) or an siRNA (siDdx5/17) targeting a conserved region shared by Ddx5 and Ddx17 (Figure 1A). Whole-transcriptome analysis was performed using Affymetrix Exon Arrays, which allowed us to assess expression at the global gene level as well as at the exon level. The median intensity of exonic probes of each gene was computed in each sample from four independent experiments. Using cutoffs of 1.5 for fold change and 0.05 for *P*-value, 354 (67%) and 173 (33%) genes were predicted to be activated and repressed by E2, respectively (Figure 1B). Comparative analysis of control and Ddx5/17-depleted cells (Supplementary Table S1) revealed that Ddx5 and Ddx17 are required for the regulation of 186 (53%) of E2-activated and 98 (57%) of E2-repressed genes (Figure 1B and Supplementary Table S1) as validated by RT–qPCR for a large number of cases (Figure 1C). Similar results were observed in a subset of selected genes using another set of siRNAs targeting Ddx5 and Ddx17 (Figure 1D and Supplementary Figure S2A).

The observed effects likely occurred at the transcriptional level as Ddx5/17 depletion abrogated E2-mediated effects at the pre-mRNA level and did not significantly affect the half-life of the tested E2-regulated mRNAs (Figure 1E and Supplementary Figure S2C). Furthermore, ERα and Ddx5 were detected on a subset of E2-target genes and their recruitment was increased after E2 treatment as demonstrated by ChIP assay (Figure 1F). These results are in agreement with previous reports (9,11,22,23) indicating that Ddx5 and Ddx17 are *bona fide* ERα transcriptional coregulators and are therefore required for mediating E2 effects on transcription. However, we observed that Ddx5/17 depletion also decreased ERα protein (but not mRNA) levels (Figure 1G), suggesting that Ddx5 and Ddx17 may also contribute to the estrogen-signaling pathway by another mechanism (see below). Of note, Ddx5/17 depletion did not affect other signaling pathways (Supplementary Figure S2D).

**Figure 2.**
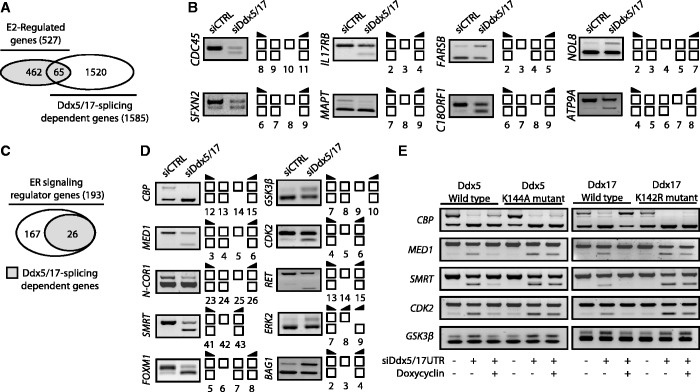
Ddx5 and Ddx17 regulate splicing of a subset of E2-target genes and genes involved in estrogen-signaling pathway. (**A**) *in silico* prediction of Exon array analysis. Venn diagram representing E2-regulated genes and genes that were regulated by Ddx5 and Ddx17 at the splicing level. (**B**) Validation by RT–PCR of splicing variants produced from E2-target genes and regulated by Ddx5 and Ddx17. Exons are represented by white boxes and primers used for PCR by black arrows. (**C**) Diagram representing ER-signaling regulators genes that were regulated by Ddx5 and Ddx17 at the splicing level. (**D**) Validation by RT–PCR of splicing events regulated by Ddx5/17. MCF-7 cells were transfected for 48 h with a Control siRNA (siCTRL) or siDdx5/17, then treated for 10 h with E2 (10 nM). Depletion of Ddx5 and Ddx17 induced both exon inclusion and exon skipping. (**E**) Rescue experiments in inducible stable cell lines. Re-expression of wild-type or mutated Ddx5 (left panel) and Ddx17 (right panel) forms in MCF-7 inducible stable cell lines after transfection with siRNAs targeting *DDX5* and *DDX17* UTRs was induced by Doxycyclin (1 µg/ml) treatment.

In addition to their role in transcription, Ddx5 and Ddx17 have been shown to play a role in alternative splicing regulation (14–17). The analysis of Exon Array data revealed that Ddx5 and Ddx17 silencing changed the splicing pattern of 65 genes among the E2-regulated genes (Figure 2A and Supplementary Table S1), as validated by RT–PCR (Figure 2B). Therefore, Ddx5 and Ddx17 are not only required for the E2-mediated effects on transcription, but also for the production of specific isoforms from, at least, a subset of endogenous E2-regulated genes. However, as most of the genes regulated at the splicing level by Ddx5 and Ddx17 were not regulated at the transcriptional level by E2 treatment (Figure 2A), this suggested that Ddx5/17 effects on splicing and E2 effects on transcription were mainly independent.

### Ddx5 and Ddx17 regulate splicing of several genes involved in the estrogen-signaling pathway

Among 193 genes encoding for mediators or regulators of the estrogen-signaling pathway, 26 were predicted to be regulated by Ddx5/17 at the splicing level (Figure 2C and Supplementary Figure S3A). For example, several genes coding for protein kinases involved in ERα phosphorylation, including CDK2, p38 MAPK (ERK2) and GSK3β, were affected at the splicing level when Ddx5 and Ddx17 were knocked down (Figure 2D and Supplementary Figure S3A). Likewise, several ERα transcriptional coregulators were affected at the splicing level by Ddx5/17 silencing (Figure 2D and Supplementary Figure S3A). This included transcriptional coactivators like CREB-binding protein (CBP) and the Mediator Subunit 1 (MED1) that plays a crucial role to anchor liganded ERα to the transcriptional pre-initiation complex (24), as well as several corepressors such as the nuclear corepressor 1 (N-CoR1) and its paralog N-COR2, also known as SMRT (Silencing Mediator of Retinoic acid and Thyroid hormone receptor) (25). The identity of some splicing variants was verified by sequencing (Supplementary Figure S4).

To further confirm the effects of Ddx5 and Ddx17 on splicing, inducible stable MCF-7 cell lines expressing wild type or helicase-mutated Ddx5 were transfected with a control siRNA or a mixture of siRNAs targeting *DDX5* and *DDX17* UTRs. The re-expression of wild-type, but not mutated Ddx5, partially rescued the effect of the siDdx5/17 UTR on the splicing pattern (Figure 2E, left panel). Similar results were obtained in inducible stable MCF-7 cell lines expressing wild-type Ddx17 or the mutant K142R lacking the helicase activity (Figure 2E, right panel). This observation raised the possibility that Ddx5 and Ddx17 contributed to the ERα-signaling pathway not only as ERα transcriptional coregulators but also by regulating the splicing pattern of key actors of the pathway.

**Figure 3.**
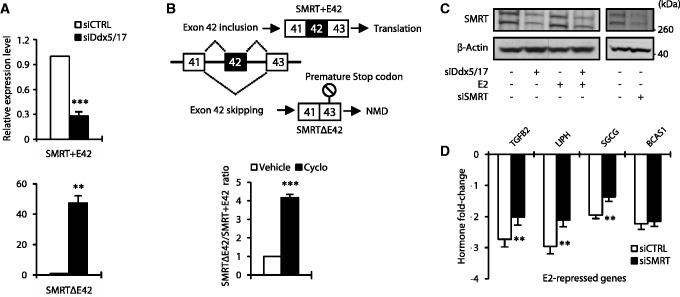
Ddx5 and Ddx17 control SMRT protein expression level. (**A**) RT–qPCR analysis measuring SMRT splicing variant expression level in MCF-7 cells transfected for 48 h with a control siRNA (siCTRL) or siDdx5/17. Specific primers were designed to amplify transcripts containing or not exon 42, SMRT + E42 and SMRTΔE42, respectively. (**B**) Upper panel, skipping of exon 42 results in a premature stop codon in exon 43. Lower panel, RT–qPCR analysis measuring SMRT splicing variant ratio in MCF-7 cells after 6 h of cycloheximide treatment (10 µg/ml). (**C**) Western blot analysis of SMRT protein expression level and β-Actin as a loading control in MCF-7 cells transfected with a control siRNA, siDdx5/17 or siRNA-targeting SMRT mRNA. (**D**) Hormone fold change induced by E2 treatment for 10 h of the expression level of a subset of mRNAs as determined by RT-qPCR in MCF-7 cells transfected with siCTRL or siSMRT. Histograms represent the average of at least three independent experiments. Error bars represent s.e.m.; **P < 0.01; ***P < 0.001 (*t* test).

To address the functional relevance of splicing regulation of key actors of the ERα-signaling pathway by Ddx5 and Ddx17, we focused on the SMRT transcriptional corepressor. As shown in Figure 3A, RT–qPCR analysis confirmed the RT–PCR data (Figure 2D) and showed that Ddx5/17 depletion induced exon 42 skipping (SMRTΔE42). Interestingly, exon 42 skipping is predicted to introduce a premature stop codon in exon 43 (Figure 3B, upper panel), suggesting that SMRTΔE42 transcripts might be unproductive (that is, not translated) and regulated by the nonsense-mediated mRNA decay (NMD) pathway. Supporting this hypothesis, incubation of MCF-7 cells with the translation inhibitor cycloheximide, that blocks mRNA degradation by the NMD pathway, increased the ratio of SMRTΔE42 transcripts to transcripts containing exon 42 (SMRT + E42) (Figure 3B, lower panel). In addition and as expected from the increased NMD-degraded SMRTΔE42 production, Ddx5/17 depletion induced a decrease of SMRT protein expression levels (Figure 3C, left panel). Finally, MCF-7 cells transfected with siRNA targeting SMRT mRNA showed a significantly reduced E2 effect on a subset of downregulated transcripts compared with control cells (Figure 3C, right panel and 3D). Altogether, these data indicated that the role of Ddx5 and Ddx17 in the regulation of the estrogen-signaling pathway is not limited to their function as ER transcriptional coregulators on estrogen-regulated genes, but also pointed to a previously undocumented regulation of coregulated splicing events directly linked to this pathway.

### Ddx5 and Ddx17 play a major role in the androgen-signaling pathway

**Figure 4.**
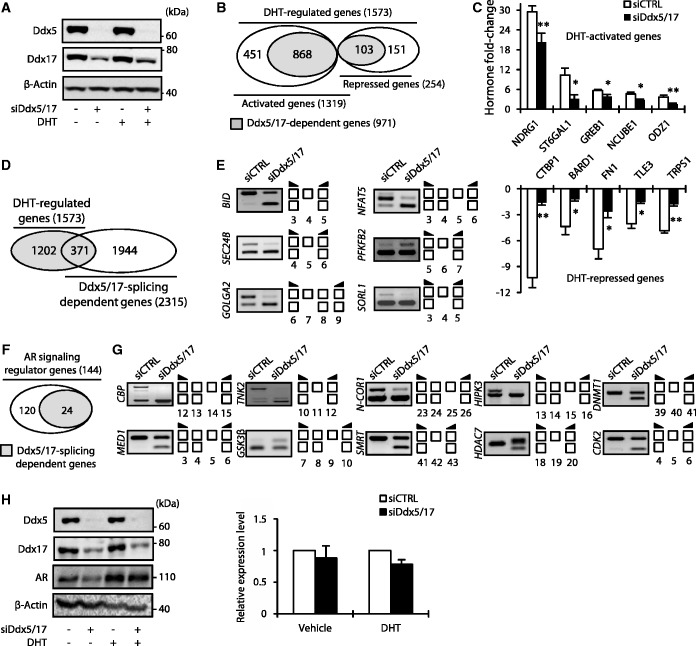
Ddx5 and Ddx17 play a major role in the androgen-signaling pathway. (**A**) Western blot analysis of Ddx5, Ddx17 and β-Actin as loading control, using total protein extract from LNCaP cells transfected with either a control siRNA (siCTRL) or siDdx5/17 in the presence or absence of DHT for 24 h. (**B**) *In silico* prediction of exon array analysis of genes regulated at the global expression level by DHT and dependent of Ddx5 and Ddx17 (in gray). (**C**) Hormone fold change induced by DHT treatment for 24 h of the expression level of a subset of mRNAs as determined by RT–qPCR in LNCaP cells transfected with siCTRL or siDdx5/17 (genes upregulated by DHT in the upper panel and genes downregulated by DHT in the lower panel). (**D**) *In silico* prediction of Exon array analysis. Venn diagram representing DHT-regulated genes and genes that were regulated by Ddx5 and Ddx17 at the splicing level. (**E**) Validation by RT–PCR of splicing variants produced from DHT-target genes and regulated by Ddx5 and Ddx17. LNCaP cells were transfected with siCTRL or siDdx5/17 then treated by DHT (10 nM) for 24 h. (**F**) Diagram representing AR-signaling regulators genes that were regulated by Ddx5 and Ddx17 at the splicing level. (**G**) Validation by RT–PCR of splicing events regulated by Ddx5/Ddx17 in the same experimental condition than F panel. (**H**) Western blot analysis of Ddx5, Ddx17, AR proteins level. β-Actin was used as loading control (left panel). LNCaP cells were transfected with either a control siRNA or siDdx5/17 in the presence or absence of DHT for 24 h. RT–qPCR analysis of the relative expression level of AR mRNA (right panel). Histograms represent the average of at least three independent experiments. Error bars represent s.e.m.; *P < 0.05; **P < 0.01 (*t*-test).

As for ERα, Ddx5 and Ddx17 are transcriptional coregulators of AR. To study the genome-wide contribution of Ddx5 and Ddx17 to the androgen-signaling pathway, we applied the same experimental strategy using the human LNCaP prostate cancer cell line that was treated with DHT for 24 h (Figure 4A). Affymetrix Exon array analysis predicted that out of a total of 1573 DHT-regulated genes, 1319 (84%) were activated and 254 (16%) repressed (Figure 4B). *In silico* analysis predicted that 868 (66%) of DHT-activated and 103 (40%) of DHT-repressed genes were regulated in a Ddx5/17-dependent manner (Figure 4B and Supplementary Table S2), as validated by RT–qPCR (Figure 4C).

When analyzing Exon Array data at the exon level, we identified 371 DHT-regulated genes (among 1573) whose splicing was affected by Ddx5/17 depletion (Figure 4D and Supplementary Table S2), as validated by RT–PCR (Figure 4E). Finally, as shown in Figure 4F, several (24 out of 144) Ddx5/17-regulated splicing variants are produced from genes involved in the regulation of the androgen-signaling pathway (Figure 4G and Supplementary Figure S3B).

Of particular interest, and similarly to our observation on ERα, Ddx5/17 depletion in LNCaP cells, reduced AR protein (but not mRNA) level (Figure 4H). Therefore, Ddx5 and Ddx17 likely contribute to both estrogen and androgen-signaling pathways through several mechanisms.

### Ddx5 and Ddx17 stabilize ERα and AR by modulating GSK3β splicing

**Figure 5.**
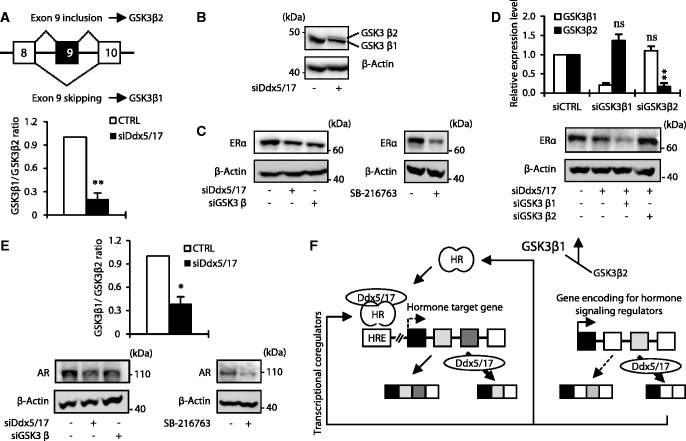
Ddx5 and Ddx17 stabilize ERα and AR protein by modulating GSK3β splicing. (**A**) Schematic representation of GSK3β primary transcripts (upper panel). RT–qPCR analysis measuring the GSK3β1 and GSK3β2 ratio in MCF-7 cells transfected with a control siRNA or siDdx5/17. (**B**) Western blot analysis of GSK3β and β-actin as a loading control in MCF-7 cells transfected with a control siRNA or siDdx5/17. (**C**) Western blot analysis of ERα protein level and β-actin as a loading control in MCF-7 cells transfected with a control siRNA, siDdx5/17 or siGSK3β (left panel) or treated with 20 µM of the SB-216673 GSK3β inhibitor for 24 h (right panel). (**D**) RT–qPCR analysis measuring GSK3β1 and GSK3β2 splicing variant expression level in MCF-7 cells transfected with specific siRNAs targeting each isoform (i.e. siGSK3β1 and siGSK3β2, upper panel). Western blot analysis of ERα protein level and β-Actin as a loading control in MCF-7 cells transfected with a control siRNA, siDdx5/17 and siGSK3β1 or siGSK3β2 (lower panel). (**E**) RT–qPCR analysis measuring the GSK3β1 and GSK3β2 ratio in LNCaP cells transfected with a control siRNA or siDdx5/17 (upper panel). Western blot analysis of AR protein level and β-actin as a loading control in LNCaP cells transfected with a control siRNA, siDdx5/17 or siGSK3β or treated with 20 µM of the SB-216673 GSK3β inhibitor for 24 h (lower panels). (**F**) Ddx5 and Ddx17 that are recruited by Steroid Hormone Receptors (HR) on Hormone Responsive Elements (HRE) act as transcriptional coregulators and regulate alternative splicing of a least a subset of hormone-regulated genes (left panel). Meanwhile, Ddx5 and Ddx17 control the expression at the splicing level of key regulators of the steroid hormone-signaling pathways. Effectors of the steroid hormone-signaling pathways regulated at the splicing level by Ddx5/17 can either be transcriptional coregulators (e.g. SMRT) or proteins modulating HR post-translational modifications, like GSK3β that controls AR and ERα protein expression levels.

Comparing the data obtained in both MCF-7 and LNCaP cells, we observed that several genes regulated at the splicing level by Ddx5/17 are involved in both ERα and AR-signaling pathways (e.g. CBP, MED1, N-COR1, SMRT and GSK3β) (Figure 2D, 4G and Supplementary Figure S3). Interestingly, it has been shown that the GSK3β kinase phosphorylates and stabilizes both ERα (5,26) and AR (6,27). Two GSK3β isoforms, resulting from exon 9 alternative splicing, have been reported in rodents (28,29) and human (30). The conventional isoform GSK3β1 that regulates ERα and AR protein stability does not contain exon 9 in contrast to the GSK3β2 isoform (Figure 5A, upper panel). Interestingly, exon 9 encodes for a 13 amino acid peptide within the kinase catalytic domain, which reduces the kinase activity of the GSK3β2 isoform compared with the canonical GSK3β1 isoform (28,30,31).

We first validated the effect of Ddx5/17 depletion on GSK3β exon 9 splicing by RT–qPCR analysis (bottom panel of Figure 5A). This confirmed that in the absence of Ddx5/17, exon 9 inclusion augments, indicating that the production of GSK3β2 splicing variant is increased while the GSK3β1 splicing variant is decreased in MCF-7 cells. Accordingly, a western blot analysis showed that Ddx5/17 silencing resulted in the appearance of a slower migrating protein band detected by the GSK3β antibody (Figure 5B), consistent with the detection of the GSK3β2 isoform (32), while the GSK3β1 isoform decreased.

As previously reported (5,26,33), GSK3β depletion and pharmacological inhibition resulted in ERα protein level decrease (Figure 5C). Remarkably, Ddx5/17 depletion had the same effect (Figure 5C) and this effect was further enhanced in MCF-7 cells transfected with specific siRNAs (Figure 5D, upper panel) targeting the GS3K3β1 but not the GSK3β2 isoform (Figure 5D, bottom panel). Collectively these results support a model in which Ddx5/17 silencing reduces the production of the canonical GSK3β1 isoform, resulting in a decreased ERα protein level. Similar results were obtained in LNCaP cells: Ddx5/17 depletion decreased the production of the conventional GSK3β1 isoform (Figure 5E, upper panel) as well as AR protein level, as did GSK3β depletion or pharmacological inhibition (Figure 5E, bottom panel), as previously reported (6,27,34,35).

Therefore, Ddx5/17 directly participate to the estrogen- and androgen-signaling pathways as ERα and AR transcriptional coregulators but also indirectly by regulating alternative splicing of other transcriptional coregulators or other actors of the signaling pathways (Figure 5F, and see ‘Discussion’ section).

## DISCUSSION

Activation of the estrogen and androgen-signaling pathways requires the concerted action of a plethora of factors that either affect post-translational modifications of ERα and AR or are recruited on hormone target genes to mediate their transcriptional effects. In this work, we report that the Ddx5 and Ddx17 RNA helicases, that have been shown to be ERα and AR transcriptional coregulators (see ‘Introduction’ section), are master regulators of the estrogen and androgen-signaling pathways by controlling transcription and splicing both upstream and downstream of receptors (Figure 5F). Indeed, Ddx5 and Ddx17 regulate the expression of a large number of estrogen- and androgen-target genes both at the transcriptional and splicing levels (Figures 1–4). Therefore, Ddx5 and Ddx17 intervene downstream of the signaling pathways for the transcriptional and splicing regulation of hormone-targeted genes. This result observed on a large scale on endogenous hormone target genes supports a model where at least some transcriptional coregulators are not only involved in the quantitative regulation of endogenous gene expression but also in their qualitative regulation by allowing the production of specific splicing variants in response to stimuli.

Furthermore, we show that Ddx5 and Ddx17 act upstream of the estrogen and androgen-signaling pathways by controlling the expression, at the splicing level, of several key regulators of the hormone-signaling pathways. (Figures 1–4 and 5F). Of particular interest, we showed that Ddx5 and Ddx17 controlled the expression level of the SMRT transcriptional coregulator. Indeed, Ddx5/17 depletion favored the production of a SMRT splicing variant that is degraded by the NMD pathway, leading to the decrease of SMRT protein level (Figure 3). Furthermore, we demonstrated that Ddx5 and Ddx17 regulated alternative splicing of GSK3β, thereby controlling AR and ERα protein levels (Figure 5). Thus, our data show that Ddx5/17 control steroid hormone-signaling pathways on a large scale, by acting both upstream and downstream of hormone receptors.

One interesting remaining question is whether Ddx5 and Ddx17 effects on transcription and splicing are somehow connected. At the transcriptional level, it has been proposed that Ddx5 and Ddx17 are recruited at the promoter level by transcriptional factors and serve as a bridge between transcription factors and other transcriptional coregulators, in particular, histone modifiers CBP (36,37) and HDACs (38). The effects of Ddx5 and Ddx17 in splicing are less characterized. It has been proposed that, these proteins may help the binding of splicing regulators on their target pre-mRNAs owing to their RNA helicase activity (14,15,39). Interestingly, Ddx5 and Ddx17 interact with RNA polymerase II (RNAPII) and could affect transcription elongation (40,41). Because transcription elongation can have an impact on alternative splicing regulation (42), Ddx5 and Ddx17 could impact on splicing owing to their effects on transcription elongation. However, as most of the genes regulated at the splicing level by Ddx5 and Ddx17 were not regulated at the transcriptional level by E2 or DHT treatment (Figures 2A and 4D), this suggested that Ddx5/17 effects on splicing and E2 or DHT effects on transcription were mainly independent. However, this does not exclude a link between transcription and splicing. For example, Ddx5 and Ddx17 may have to be first recruited by the transcriptional machinery before being loaded onto the nascent RNA molecules to impact on splicing; the recruitment by the transcriptional machinery may not necessarily be measurable by an effect on gene transcription activity.

Even though further experiments are required to better understand the mechanisms by which Ddx5 and Ddx17 affect transcription and splicing, their broad effects on the estrogen and androgen-signaling pathways has several physio-pathological consequences. First, the misregulation of Ddx5 and/or Ddx17 expression level and/or activity owing to posttranslational modifications that was reported in many cases of breast and prostate cancer (10,23,43–45), may have a broad impact on the estrogen- and androgen-signaling pathways that play a major role in the etiology and progression of those cancers. For example, the *Ddx5* gene is fused in frame to the ETV4 gene in prostate tumors (43). Based on our data, it can be anticipated that such a translocation may have a broad impact on the androgen-signaling pathway in these tumors.

Second, we observed that many of the Ddx5/17 splicing-regulated genes have been reported to play a direct role in tamoxifen resistance, one of the major endocrine therapies of breast cancer. This includes several transcriptional coregulators such as SMRT, MED1 and NCoR1 involved in resistance to tamoxifen (46–48) and several kinases such as GSK3β, CDK2 and RET which participate, directly or indirectly, to ERα phosphorylation and thus, the hormone-independent activation of ER and tamoxifen resistance (49–51). Interestingly as well, Ddx5 expression correlates with that of HER-2/neu epidermal growth factor receptor (23,45). It has been reported that overexpression of HER2/neu is associated with increased ER phosphorylation on ser-118 residue (52) which could play a role in resistance to tamoxifen by permanent ERα activation. Interestingly, even though further experiments are required because of potential contradictory reported data, high Ddx5 expression level could be associated with higher tumor grade or poor prognosis in ER-positive breast cancer patients (23,53). Furthermore, a recent report indicate that, Ddx5 and Ddx17 proteins level change significantly according to breast cancer subtype (45) and is highly dependent on certain miRNAs abundance and regulation, in particular miR-206 that has been reported to play a major role in breast cancer and estrogen pathway (54). Therefore, changes in the expression of Ddx5 and Ddx17, in particular under the control of altered miRNAs in breast cancer, could play a role in resistance to tamoxifen, in particular owing to their effects on splicing of genes involved in resistance (see above). It would be particularly interesting to test whether resistance to endocrine therapy is associated with mis-regulation of splicing of genes involved in steroid hormone-signaling pathways.

While many researches have been concentrated on the canonical GSK3β1, ubiquitously distributed in organs, minor attention was attributed to GSK3β2 isoform, whose substrate preference and physiological significance remain unclear. In GSK3β2 long isoform, exon 9 translation produces a 13 amino acid insert of unknown function in an external loop near the catalytic domain (28–30) which was associated with reduced kinase activity (30,31). Changes in the balance of GSK3β2/GSK3β1 isoforms due to alternative splicing regulation could have a broad impact in cancer initiation or progression, not only owing to an effect on ER and AR protein level, as reported in this work but also owing to the key role of GS3Kβ in the β-catenin homeostasis and its cross-talk with WNT-signaling pathway.

Because there is increasing evidence that the interplay between transcription and splicing participates in signaling pathway regulation and outcomes, we created a freely available website, named SSAS-DB (http://fasterdb.lyon.unicancer.fr/ssas-db/home.pl) that provides genome-wide information on the impact of estrogen, androgen and Ddx5/17 on gene expression in breast and prostate cancer cell lines, at both whole-gene (transcriptional) and exon- (alternative splicing) levels. This website is a valuable tool to analyze the splicing variants of hormone-regulated genes or genes involved in hormone-signaling pathways (see Supplementary Figure S5 for further information on SSAS-DB).

In conclusion, our work provides a new understanding of steroid hormone-signaling pathways owing to the identification of two related proteins, Ddx5 and Ddx17, that not only mediate the regulation at both transcriptional and splicing levels of a large subset of steroid hormone target genes, but also control a set of splicing events in genes that, in turn, control steroid receptor abundance and activity. Thus, we propose that Ddx5 and Ddx17 are master regulators of estrogen and androgen-signaling pathways by acting both upstream and downstream of receptors.

## SUPPLEMENTARY DATA

Supplementary Data are available at NAR Online, including [55].

## FUNDING

The Institut National du Cancer (INCa); Agence Nationale de la Recherche (ANR); Fondation Recherche Médicale (FRM); Ligue Nationale Contre le Cancer (to S.S. and E.D.); Association Française contre les Myopathies (to M.P.E.), FRM (to E.Z.) and Association pour la Recherche sur le Cancer (to S.G.). Funding for open access charge: Institut National du Cancer (INCa) Agence Nationale de la Recherche (ANR) Fondation Recherche Médicale (FRM) Ligue Nationale Contre le Cancer (LNCC) Association Française contre les Myopathies (AFM) Association pour la Recherche sur le Cancer (ARC)

*Conflict of interest statement*. None declared.

## Supplementary Material

Supplementary Data
