# Supplementary material for: The Ddx5 and Ddx17 RNA helicases are cornerstones in the complex regulatory array of steroid hormone-signaling pathways
Source: Nucleic Acids Res. 2013 Nov 25;42(4):2197–207. doi: 10.1093/nar/gkt1216 (PMC3936752; doi:10.1093/nar/gkt1216)
Supplement: Supplementary Data [file supp_42_4_2197__index.html]

The Ddx5 and Ddx17 RNA helicases are cornerstones in the complex regulatory array of steroid hormone-signaling pathways — The Ddx5 and Ddx17 RNA helicases are cornerstones in the complex regulatory array of steroid hormone-signaling pathways — Supplementary Data 

# The Ddx5 and Ddx17 RNA helicases are cornerstones in the complex regulatory array of steroid hormone-signaling pathways

## Supplementary Data

files

**Files in this Data Supplement:**

- Supplementary Data - pdf file
- Supplementary Data - xlsx file
