# Supplementary material for: The Ddx5 and Ddx17 RNA helicases are cornerstones in the complex regulatory array of steroid hormone-signaling pathways
Source: Nucleic Acids Res. 2013 Nov 25;42(4):2197–207. doi: 10.1093/nar/gkt1216 (PMC3936752; doi:10.1093/nar/gkt1216)
Supplement: Supplementary Data [file supp_gkt1216_nar-02549-y-2013-File007.pdf]

Supplementary Fig. S1

Identification of hormone-regulated Ddx5/Ddx17 dependent genes

Algorithm 1

Hormone-activated genes :
Hormone fold-change (B/A) > 1.5

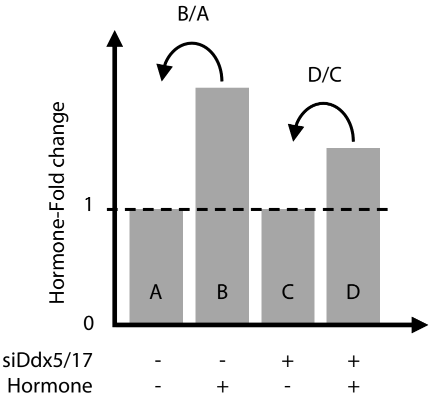

Hormone-repressed genes :
Hormone fold-change (B/A) < -1.5

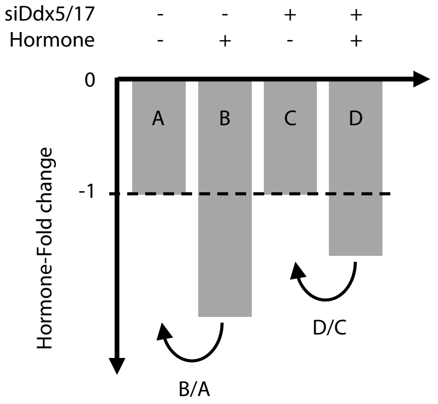

Gene is dependent on Dddx5/17 for hormone regulation if (B/A)/(D/C) ratio >1.2 & *P* value <0.05

Algorithm 2

Hormone-activated genes :
Hormone fold-change (B/A) > 1.5

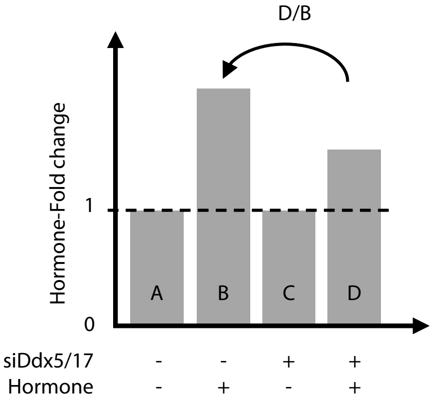

Hormone-repressed genes :
Hormone fold-change (B/A) < -1.5

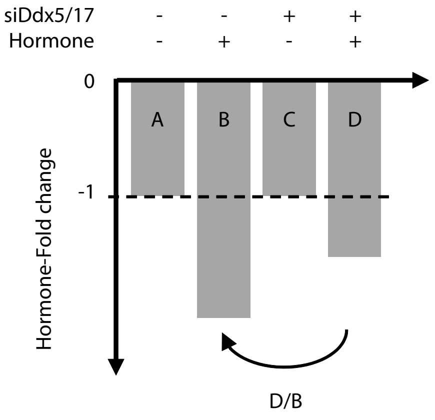

Gene is dependent on Dddx5/17 for hormone regulation if :

D/B ratio < -1.5 & D<B
P value <0.05

D/B ratio > 1.5 & D>B
P value <0.05

Fig. S1
Analytical strategies of high throughput exon array data
Hormone fold-change (HFC) was computed either in the presence (B/A ratio) or in the absence (D/C ratio) of Ddx5/17. Hormone-regulated gene is considered to be dependent on Ddx5/17 when HFC was significantly decreased with a threshold higher than 1.2 and *P* value <0.05 (algorithm 1). Algorithm 2 (lower panel) allows determining the genes that are regulated in a Ddx5/17 dependent manner if the global expression level under hormone treatment was significantly reduced in Ddx5/17-depleted cells when compared to hormone effect in control cells (D/B ratio).

Supplementary Fig. S2

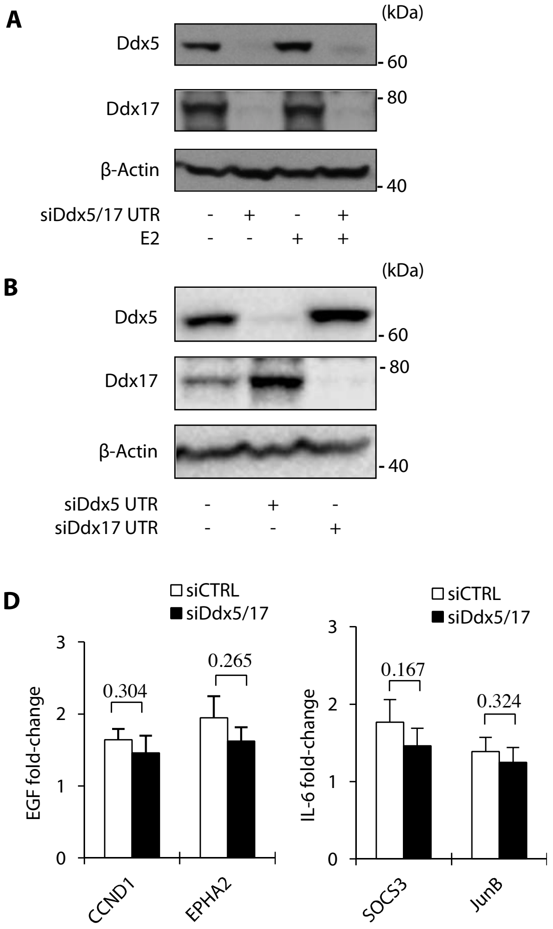

C

mRNA half-life in vehicle-treated MCF-7 cells

	siCTRL		siDdx5/17	
GLA	2,32	(±0,36)	3,02	(±0,11)
TMEM26	1,86	(±0,28)	2,15	(±0,29)
STC1	1,99	(±0,14)	2,83	(±0,36)
IL20	1,25	(±0,16)	1,58	(±0,18)
TGFβ2	1,99	(±0,13)	2,73	(±0,15)
LIPH	2,11	(±0,15)	2,62	(±0,10)
SGCG	2,40	(±0,28)	3,75	(±1,23)
BCAS1	2,80	(±0,69)	2,43	(±0,60)

mRNA half-life in Estradiol-treated MCF-7 cells

	siCTRL		siDdx5/17	
GLA	1,85	(±0,31)	2,27	(±0,10)
TMEM26	1,35	(±0,23)	1,68	(±0,12)
STC1	2,47	(±0,58)	2,74	(±0,53)
IL20	1,26	(±0,25)	1,41	(±0,15)
TGFβ2	4,70	(±0,91)	3,18	(±0,39)
LIPH	3,27	(±0,66)	2,59	(±0,16)
SGCG	3,52	(±0,52)	3,96	(±0,39)
BCAS1	3,06	(±0,50)	3,97	(±0,93)

Fig. S2

Ddx5 and Ddx17 do not affect E2-target transcripts half-life

(A) Western blot analysis of Ddx5, Ddx17. β-Actin was used as loading control, MCF-7 were transiently transfected with Control siRNA (siCTRL) or a mixture of siRNAs targeting Ddx5 and Ddx17 UTRs (siDdx5/17 UTR).

(B) Western blot analysis of Ddx5, Ddx17. β-Actin was used as loading control, MCF-7 were transiently transfected with Control siRNA (siCTRL) or with siRNAs specific targeting Ddx5 orDdx17 UTRs separately. Of note, depletion of Ddx5 increased Ddx17 protein level and Ddx5 global level is increased in Ddx17-depleted MCF-7 cells as previously reported (Supplementary reference 55)

(C) E2-regulated mRNAs half-life. MCF-7 cells transfected with a control siRNA (siCTRL) or siDdx5/17 were incubated with Actinomycin D (5 µg/ml) for 3, 6 or 9 hours either in the absence (upper panel) or the presence of E2 (lower panel). Mature mRNA levels were measured by RT-qPCR. Data are represented as the mean of at least three independent experiments. mRNAs half-life were calculated as X (±s.e.m.) hour in control and Ddx5/17-depleted cells, respectively.

(D) Relative fold change induced by EGF (100 ng/mL, left panel) or IL-6 (20 ng/mL, right panel) treatment for 12 hours on the expression level of target genes as determined by RT-qPCR in MCF-7 cells transfected with siCTRL or siDdx5/17.

Supplementary reference 55:

Geissler, V., Altmeyer, S., Stein, B., Uhlmann-Schiffler, H. and Stahl, H. (2013) The RNA helicase Ddx5/p68 binds to hUpf3 and enhances NMD of Ddx17/p72 and Smg5 mRNA. Nucleic acids research.

Supplementary Fig. S3

A

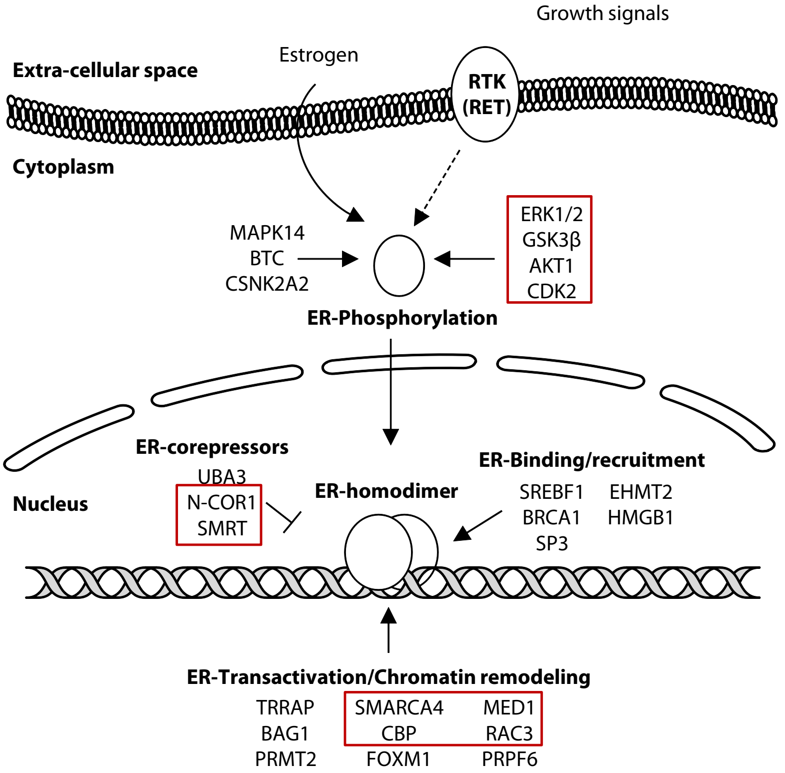

B

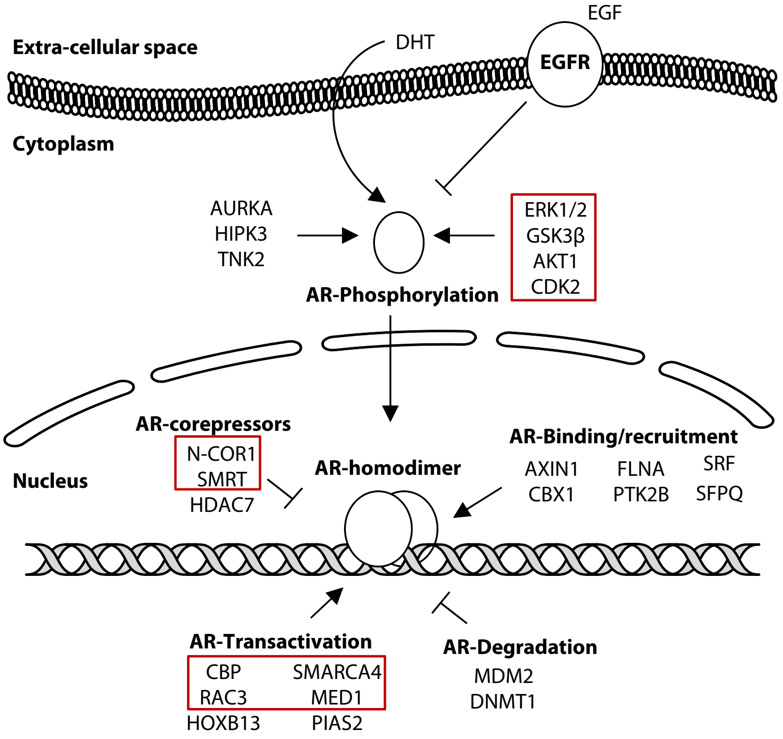

Fig. S3

Ddx5 and Ddx17 regulate alternative splicing of numerous key regulators of steroid hormone-signaling pathways

(A) Schematic representation of Estrogen signaling pathway; genes affected by Ddx5/17 depletion at their splicing level are indicated .
(B) Schematic representation of Androgen signaling pathway; genes affected by Ddx5/17 depletion at their splicing level are indicated .
Common genes that are reported to be involved in the regulation of both Estrogen and Androgen signaling pathways were highlited with red boxes.

Supplementary Fig. S4

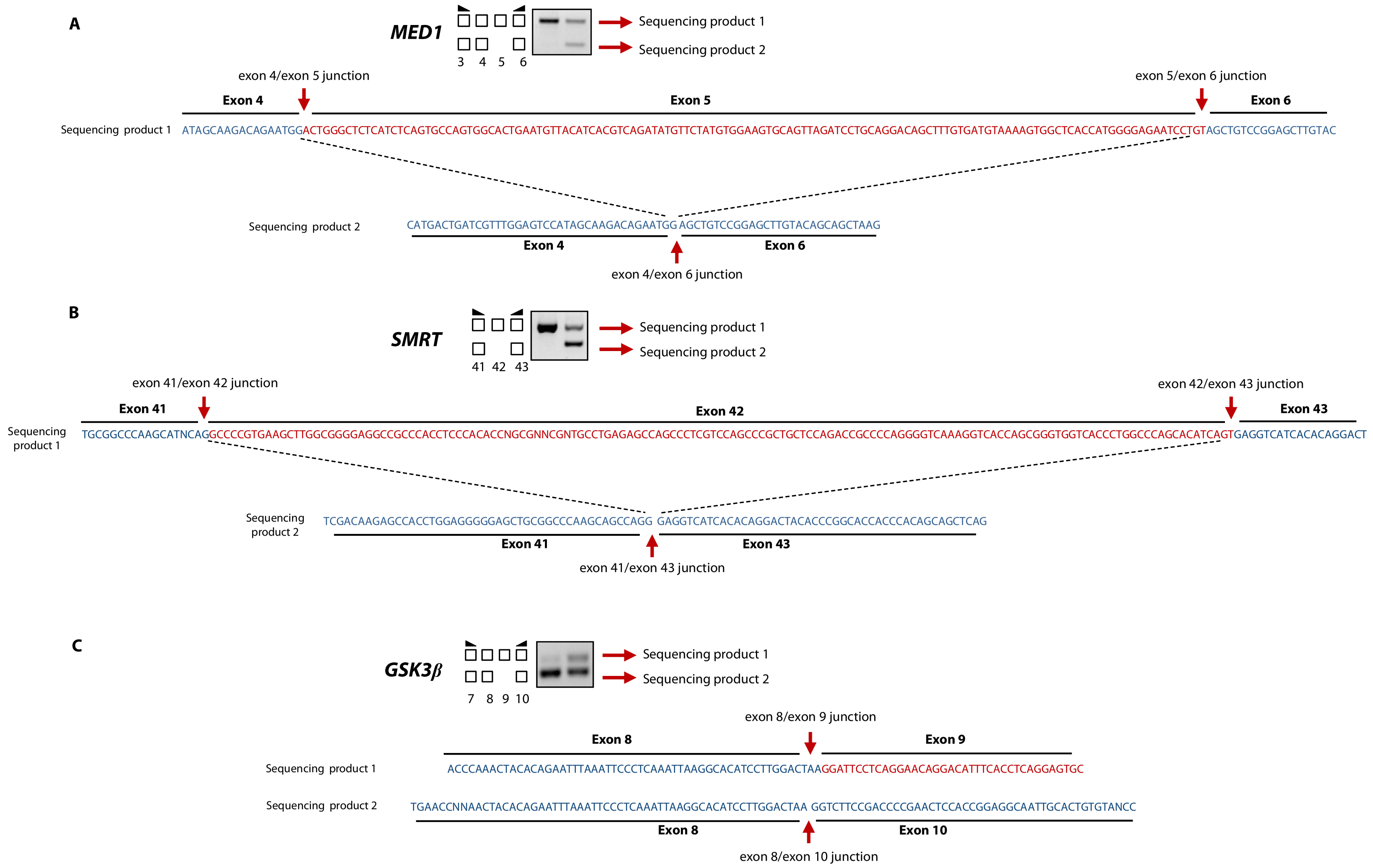

Fig. S4

Sequencing results of some Ddx5/17-splicing dependent events

MCF-7 cells were transfected for 48h with a Control siRNA (siCTRL) or siDdx5/17. PCR products were purified after agar-gel separation and sequenced. Alignment of sequences obtained from each PCR product is shown. Sequence of alternative exons are depicted in red.

(A) *MED1* gene exon 5 skiping .
(B) *SMRT* gene exon 42 skiping.
(C) *GSK3β* gene exon 9 inclusion.

Supplementary Fig. S5

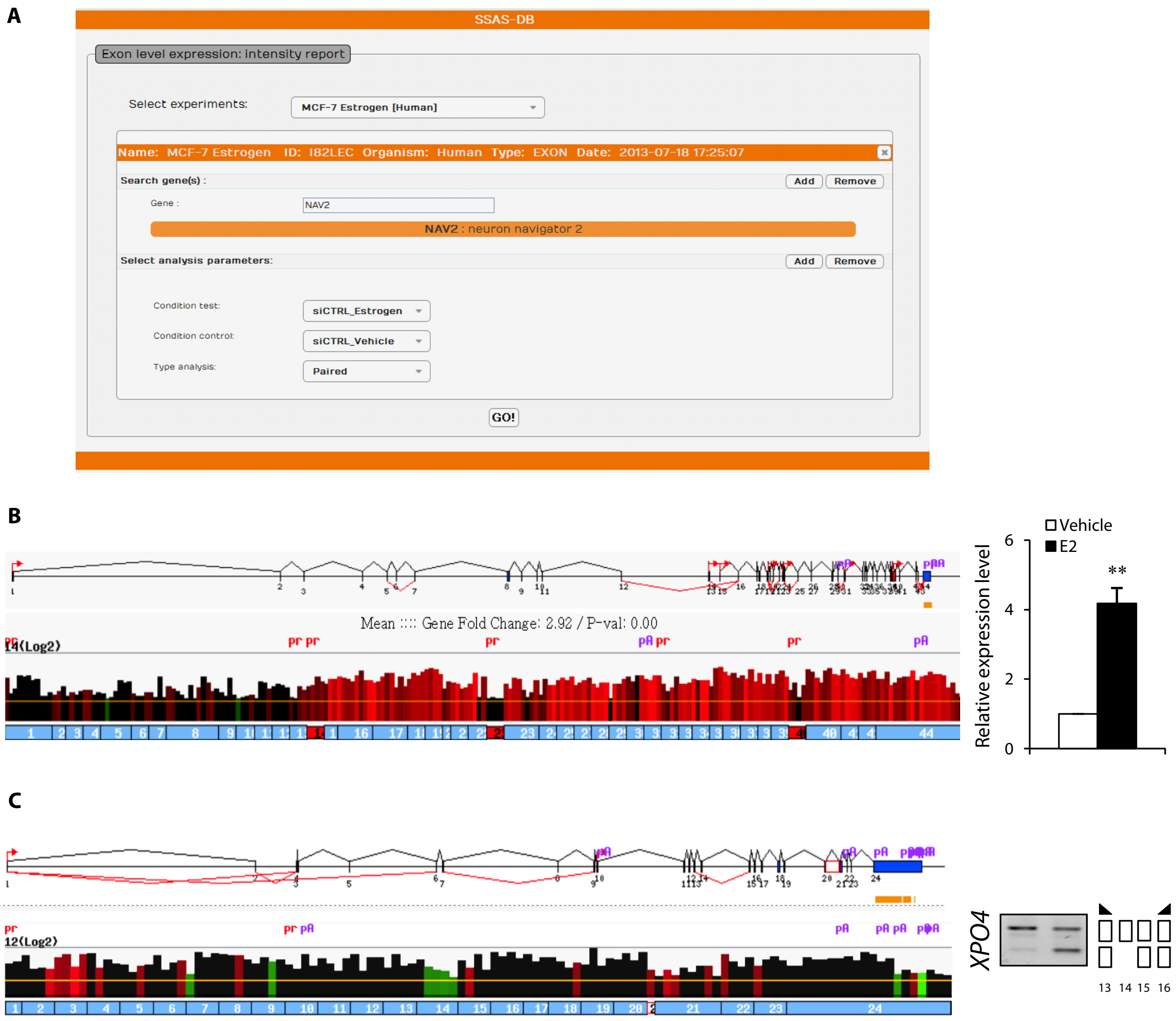

Fig. S5

SSAS-DB (Steroid Signaling and Alternative Splicing-Data-Base) web interface

(A) SSAS-DB search engine. All the datasets presented in this work are accessible in a user friendly web interface (http://fasterdb.lyon.unicancer.fr/ssas-db/home.pl) that provides exon-level informations (gene structure, alternative promoters, alternative exons and alternative polyadenylation sites) on the expression of 19.000 human genes in breast and prostate cancer cell lines in response to estrogen or androgen treatment, as well as following Ddx5/17 depletion. SSAS-DB allows users to gain insight into sex steroid hormone signaling pathways at the exon-level resolution. SSAS-DB can be interrogated using official gene symbol as illustrated for the *NAV2* gene after selecting a specific set of experiments (e.g., estradiol treatment in MCF-7 cells).

(B) Left panel, *NAV2* gene structure and Exon Array visualization module when comparing estradiol treated MCF-7 cells to vehicle treated control cells. Visualization of Affymetrix Exon Array presents probe intensities when comparing test experiments (e.g., estradiol treatment) to control experiments (i.e., vehicle treatment). Each probe is represented by a bar whose height indicates the intensity (on a log2 scale) and whose color reflects its differential expression status (on a traditional red-green scale); red probes indicate higher intensity in the test experiment while green probes indicate lower probe intensity when compared to the control experiment. Black probes point to no difference between both conditions. Probe level information is also available as tables to complement the graphical representation. Of note, estradiol treatment activates *NAV2* gene expression from exon 14 to exon 44. Remarkably, exon 14 is marked as an alternative promoter (red arrow above exon 14 on the gene structure) suggesting that estradiol treatment activates this internal promoter. Right panel, RT-qPCR quantification of *NAV2* relative mRNA level in control and E2-treated cells.

(C) *XPO4* gene structure and Exon Array visualization module when comparing Ddx5/17 depleted MCF-7 cells to cells transfected with a control siRNA (left panel). Probes targeting *XPO4* gene exon 14 are green when comparing Ddx5/17-depleted MCF-7 cells to control cells suggesting that Ddx5/Ddx17 knocking-down induced *XPO4* exon 14 skipping. Right panel, RT-PCR analysis of *XPO4* exon 14 splicing in control and Ddx5/17-depleted MCF-7 cells.

Histograms represent the average of at least three independent experiments. Error bars represent s.e.m.; ** P <0.01 (*t* test).
